# Maternal anxiety and infants' hippocampal development: timing matters

**DOI:** 10.1038/tp.2013.79

**Published:** 2013-09-24

**Authors:** A Qiu, A Rifkin-Graboi, H Chen, Y-S Chong, K Kwek, P D Gluckman, M V Fortier, M J Meaney

**Affiliations:** 1 Department of Bioengineering, National University of Singapore, Singapore; 2 Clinical Imaging Research Centre, National University of Singapore, Singapore; 3 Singapore Institute for Clinical Sciences, the Agency for Science, Technology and Research, Singapore; 4 KK Women's and Children's Hospital (KKH), Singapore; 5 Department of Obstetrics & Gynaecology, Yong Loo Lin School of Medicine, National University of Singapore, National University Health System, Singapore; 6 Department of Maternal Fetal Medicine, KK Women's and Children's Hospital, Singapore; 7 Liggins Institute, University of Auckland, Auckland, New Zealand; 8 Department of Diagnostic and Interventional Imaging, KK Women's and Children's Hospital (KKH), Singapore; 9 Departments of Psychiatry and Neurology & Neurosurgery, McGill University, Montreal, Canada

**Keywords:** antenatal anxiety, hippocampus, magnetic resonance imaging, neonatal brain, postnatal anxiety

## Abstract

Exposure to maternal anxiety predicts offspring brain development. However, because children's brains are commonly assessed years after birth, the timing of such maternal influences in humans is unclear. This study aimed to examine the consequences of antenatal and postnatal exposure to maternal anxiety upon early infant development of the hippocampus, a key structure for stress regulation. A total of 175 neonates underwent magnetic resonance imaging (MRI) at birth and among them 35 had repeated scans at 6 months of age. Maternal anxiety was assessed using the State-Trait Anxiety Inventory (STAI) at week 26 of pregnancy and 3 months after delivery. Regression analyses showed that antenatal maternal anxiety did not influence bilateral hippocampal volume at birth. However, children of mothers reporting increased anxiety during pregnancy showed slower growth of both the left and right hippocampus over the first 6 months of life. This effect of antenatal maternal anxiety upon right hippocampal growth became statistically stronger when controlling for postnatal maternal anxiety. Furthermore, a strong positive association between postnatal maternal anxiety and right hippocampal growth was detected, whereas a strong negative association between postnatal maternal anxiety and the left hippocampal volume at 6 months of life was found. Hence, the postnatal growth of bilateral hippocampi shows distinct responses to postnatal maternal anxiety. The size of the left hippocampus during early development is likely to reflect the influence of the exposure to perinatal maternal anxiety, whereas right hippocampal growth is constrained by antenatal maternal anxiety, but enhanced in response to increased postnatal maternal anxiety.

## Introduction

Anxiety has a familial component^1^ such that children of affected parents show a significantly increased risk for emotional problems.^2, 3^ Although the precise mechanism through which vulnerability is transmitted remains unclear, there is evidence that maternal emotional well being influences the offspring. Maternal stress and the resulting negative affective states during pregnancy affect fetal physiology,^4^ associate with increased behavioral inhibition in the first two years of life even after controlling for postnatal mood^5^ and predict increased risk for childhood behavioral and emotional problems and decreased gray matter density in childhood.^6^ These effects appear to reflect the transgenerational transmission of individual differences in vulnerability for anxiety-related disorders.

Effective treatment of depression of mothers of children 7–17 years of age decreases psychological problems in the offspring suggesting effects on parent–child interactions.^7^ Indeed, mood disorders promote forms of parenting^8, 9^ that enhance stress reactivity, social withdrawal and inattention,^10, 11, 12, 13^ which in turn predict an increased risk for mood disorders. However, several development models suggest that early exposure to stress may confer resistance to stressors in later life. For instance, Lyons and Parker^14^ propose a stress-inoculation model advancing the idea that exposure to mild stress during early development promotes resilience in the face of stressful circumstances in later life. In addition, data from Ellis *et al.*^15^ support the ‘biological sensitivity to context' theory, indicating that early exposure to stress can increase the liability of responses to subsequent adversity in some individuals. The mechanism and timing for such parental influences after childbirth is unclear, which complicates models of risk as well as the design and timing of preventive interventions. Existing studies on the consequences of maternal anxiety are compromised by the fact that the relevant outcome measures, including imaging studies of neural endophenotypes, have been collected in later childhood and are thus confounded by unknown postnatal influences.

A prospective, longitudinal birth cohort (Growing Up in Singapore Towards Health Outcomes; GUSTO) study provided a unique opportunity to examine the consequences of antenatal and postnatal exposure to maternal anxiety for infant brain development in early life. We focused on the hippocampus as the hippocampus regulates the production of cortisol, a biomarker of stress. A reduction of the hippocampal volume is a key neuroanatomical feature in anxiety disorder.^16^ The right hippocampus is selectively associated with stress reactivity.^17^ We anticipated that antenatal maternal anxiety would negatively associate with hippocampal volumes of infants at birth and at 6 months of age, as well as hippocampal growth patterns in early life. Moreover, we hypothesized that higher maternal anxiety in the early postnatal stage would also exert effects on hippocampal growth, though we were uncertain whether postnatal anxiety would continue to inhibit the maturation of the infant nervous system or lead to more rapid hippocampal growth, indicating adaptation to an aversive environment. The results of this study provide, to our knowledge, the first direct analysis of the link between maternal anxiety and brain development in the first six months of life.

## Materials and methods

### Subjects

A total of 189 mothers who participated in a birth cohort study, Growing Up in Singapore Towards Healthy Outcomes (GUSTO), agreed on participating in the neuroimaging study. The GUSTO cohort consisted of pregnant Asian women attending the first trimester antenatal ultrasound scan clinic at the National University Hospital and KK Women's and Children's Hospital (KKH) in Singapore. Birth outcome and pregnancy measures were obtained from hospital records. The parents were Singapore citizens or permanent residents of Chinese, Malay or Indian ethnic background. Socioeconomic status (household income) and prenatal exposure to alcohol (regular alcohol drinking) and tobacco (regular smoking, daily exposure to smoking at home and job) were extracted from survey questionnaires conducted as a part of a scheduled appointment during pregnancy. This study only included neonates with gestational age greater or equal to 35 weeks, birth weight larger than 2000 g, and a 5-min APGAR score greater than 9. The GUSTO cohort study, including imaging procedures, was approved by the National Healthcare Group Domain Specific Review Board and the Sing Health Centralized Institutional Review Board.

### Maternal anxiety questionnaire

The State-Trait Anxiety Inventory (STAI, Form Y-2) was administered at 26 weeks of pregnancy and at 3 months after the delivery to quantify maternal prenatal and postnatal levels of anxiety. The STAI is a commonly used self-report tool for assessing anxiety and consists of two subscales (state and trait anxiety) with each containing 20 items on a 4-point rating scale. State anxiety reflects a ‘transitory emotional state or condition of the human organism that is characterized by subjective, consciously perceived feelings of tension and apprehension, and heightened autonomic nervous system activity'. State anxiety may fluctuate over time and can vary in intensity. In contrast, trait anxiety denotes ‘relatively stable individual differences in anxiety proneness' and refers to a ‘general tendency to respond with anxiety to perceived threats in the environment'. The STAI is used widely in the peripartum population. Items that assess the absence of anxiety are reverse scored. The state and trait anxiety scores can be directly computed as the sum of scores of each item and have a direct interpretation: high scores on their respective scales mean more trait or state anxiety and low scores mean less. In regards to the perinatal population, the STAI has been shown to have construct validity.^18^ The reliability of this screening scale was 0.91 for both STAI state and trait subscales assessed using Cronbach's analysis for our cohort. In this study, only the trait measures at 26 weeks of pregnancy and 3 months after the delivery were used to examine the changes between the prenatal and postnatal maternal anxiety.

### MRI acquisition

A total of 189 neonates underwent fast spin-echo T2-weighted MRI at 5–17 days of life and 42 infants among them underwent the follow-up scans at 6 months of life using a 1.5-Tesla GE scanner at the Department of Diagnostic and Interventional Imaging of the KKH. The scans were acquired when subjects were sleeping in the scanner. No sedation was used and precautions were taken to reduce exposure to the MRI scanner noise. A neonatologist was present during each scan. A pulse oximeter was used to monitor heart rate and oxygen saturation throughout the entire scans.

The imaging protocol included (1) axial fast spin-echo T2-weighted MRI (repetition time (TR)=3500 ms; echo time (TE)=110 ms; field of view (FOV)=256 × 256 mm; matrix size=256 × 256; 50 axial slices with 2.0 mm thickness); (2) coronal fast spin-echo T2-weighted MRI (TR=3500 ms; TE=110 ms; FOV=200 × 200 mm; matrix size=256 × 256; 24 coronal slices with 2.0 mm thickness). The coronal T2-weighted MRI data were acquired parallel to the anterior–posterior axis of the hippocampus and only covered the temporal lobe. Each subject obtained two acquisitions of the axial T2-weighted MRI and one acquisition of the coronal T2-weighted MRI at the baseline and follow-up. All brain scans were reviewed by a neuroradiologist (M.V.F).

### Hippocampus delineation

Within individual subjects, when possible, two T2-weighted MRI acquisitions were first rigidly aligned and averaged to increase signal-to-noise ratio. In cases where only one scan was acquired, data from one scan was used in lieu of the average axial image. The skull of the averaged axial image was removed using Brain Extraction Tool^19^ and used manual and automated segmentation of the hippocampus, as discussed below.

#### Manual delineation

**Figure 1:**
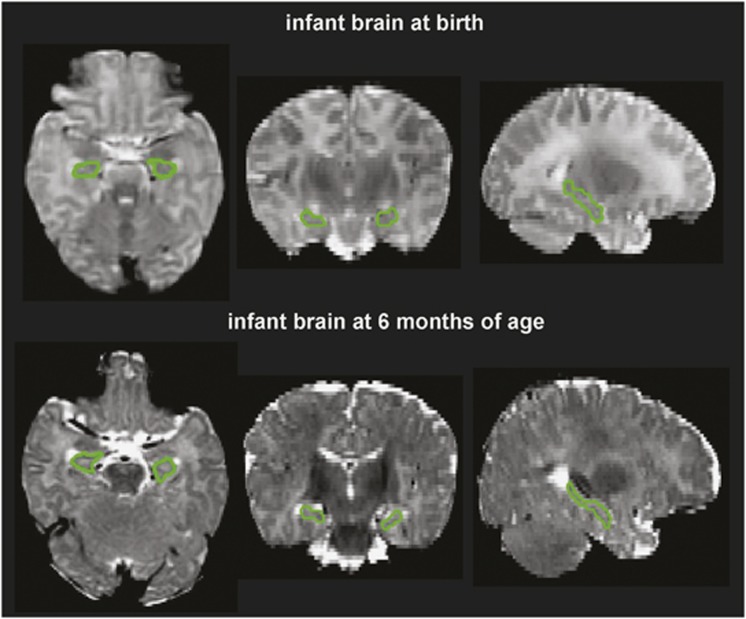
Segmented hippocampus from the brain of one infant at birth (top row) and 6 months (bottom row) of age. From left to right, the panels respectively show the axial, coronal and sagittal slices of the T2-weighted brain images.

We manually delineated the hippocampus on the image created by averaging the axial and coronal T2-weighted images within individual subjects through a halfway affine transformation. This new image had better signal-to-noise ratio and better spatial resolution than the acquired coronal and axial T2-weighted images. The manual tracing of the hippocampus was performed on the coronal view of this new image by closely following a standard protocol.^20^ MRI images of 20 neonates and 35 6-month-old infants were manually delineated. Among them, five images at each time point were manually delineated twice by one rater. Intra-class correlation coefficients were0.79 and 0.82 for the hippocampus of neonates and 6-month-old infants, respectively. Figure 1 shows examples of the manually delineated hippocampi for neonates and 6-month infants.

#### Automated segmentation for neonatal hippocampus

A Markov random field model (MRF) was used to automatically delineate the hippocampus from the neonatal T2-weighted MRI data. The mathematical model of MRF was detailed in the study by Fischl *et al.*^21^ The MRF model has been considered as one of robust automatic brain segmentation approaches because it incorporates the anatomical prior information obtained from a manually labeled training set. In our study, our training set consisted of twenty T2-weighted MRI datasets mentioned earlier. We employed the leave-one-out validation approach to evaluate the MRF segmentation accuracy for the hippocampus. The 19 images with the hippocampal manual label were used as training sets in MRF and one image with the hippocampal manual label was used as a testing set. The accuracy of the MRF segmentation for the hippocampus was 0.71, which was comparable to that of an intra-rater method as well as the recent report.^20, 21^ The volume of the hippocampus was computed as the multiplication of the number voxels in the structural mask and the image resolution.

### Statistical analysis

Multiple regression analyses first examined associations between prenatal maternal anxiety and hippocampal volumes of infants at (a) birth; and (b) six months of age; as well as (c) hippocampal growth in the first 6 months of life. Separate models evaluated each brain measure as a dependent variable. The prenatal maternal STAI trait score was entered as the primary independent variable. Relations between plausible covariates (for example, gender, birth weight, post-conceptual age on the MRI day (gestational age+days of life since birth to the MRI scan), intracranial volume, monthly household income, ethnicity, prenatal smoking exposure and prenatal alcohol exposure), and (a) predictors and (b) outcomes were assessed. When correlations reached significance with either the predictors or multiple outcomes, they were used as covariates in regression models. This process led to the inclusion of the following as covariates in regression analyses: the post-conceptual age on the MRI day, monthly household income and intracranial volume at birth. We ran the regression model with or without the postnatal maternal anxiety as an additional covariate.

Then, multiple regression analysis examined the associations of postnatal maternal anxiety with the hippocampal volumes of infants at 6 months of age as well as the growth in the hippocampal volumes in the first 6 months of infants' life. For this, the postnatal maternal STAI trait score was entered as the primary independent variable. The post-conceptual age on the MRI day (gestational age+days of life since birth to the MRI scan), monthly household income and intracranial volume at 6 months of age were entered as covariates with and without antenatal maternal anxiety as an additional covariate.

Finally, multiple regression analysis examined the associations of the difference between the prenatal and postnatal STAI trait anxiety scores with the hippocampal volumes of infants at 6 months of age as well as the growth in the hippocampal volumes in the first 6 months of infants' life. All models were adjusted for the age difference between the baseline and follow-up MRI scans, household income and growth of intracranial volume in the first 6 months of life.

## Results

### Demographics

Among 189 infants, this study excluded 5 subjects with APGAR less than 9. Through visual inspection,nine neonates had large motion in the MRI data at the baseline. Among 42 6-month infants who underwent the MRI at 6 months of age, 35 had at least one good axial T2-weighted MRI scan. Hence, the current study included 175 infants at the baseline and 35 infants at the follow-up.

Table 1 lists the demographics of mothers and infants at both baseline and follow-up. The birth weight, gestational age, gender, maternal age, prenatal alcohol and smoking exposure were comparable between the whole sample (*n*=175) and the subset of the sample with the follow-up MRI scans (*n*=35) (all P>0.05).

The pairwise correlation between measures of antenatal and postnatal maternal STAI trait anxiety scores exceeded *r*=0.50. Student's *t*-test also showed that the magnitude of the maternal anxiety level was comparable between the assessments at pregnancy and early postpartum period (*P*=0.799).

In our study, 54.45% of the 175 mothers had been exposed to smoking during pregnancy (for example, at house, at workplace and so on), whereas only nine mothers had smoking history during pregnancy. Given the known association of parental smoking with decreased birth weight and psychological problems of offsprings,^22, 23^ we further examined associations of prenatal smoking exposure with maternal anxiety and infants' hippocampal volumes using Pearson's correlation analysis. Our study did not reveal any correlation of prenatal smoking exposure with maternal anxiety (antenatal: *r*=0.120, *P*=0.141; postnatal: *r*=0.074; *P*=0.708), bilateral hippocampal volumes at birth (left: *r*=0.058, *P*=0.457; right: *r*=−0.027, *P*=0.727) and those at 6 months of life (left: *r*=0.021, *P*=0.905; right: *r*=−0.104, *P*=0.558). After removing the nine smoking mothers, the results remained unchanged.

In our study, maternal anxiety did not vary as a function of infant gender (antenatal: *r*=0.074, *P*=0.356; postnatal: *r*=0.130, *P*=0.456). Likewise, no gender differences were found in bilateral hippocampal volumes at birth (left: *r*=−0.130, *P*=0.086; right: *r*=−0.131, *P*=0.084) nor at 6 months of life (left: *r*=−0.252, *P*=0.143; right: *r*=−0.307, *P*=0.072).

Further examination of the variables listed in Table 1, via Pearson's correlation, revealed that maternal anxiety was negatively correlated only with monthly household income (antenatal: *r*=−0.341, *P*<0.001; postnatal: *r*=−0.397, *P*=0.024), suggesting that higher the monthly household income lower the maternal anxiety. Bilateral hippocampal volumes at birth were correlated with post-conceptual age on the MRI day (left: *r*=0.163, *P*=0.032; right: *r*=0.151, *P*=0.047) and intracranial volume at birth (left: *r*=0.494, *P*<0.001; right: *r*=0.566, *P*<0.001). These hippocampal volumes were also correlated with birth weight (left: *r*=0.184, *P*=0.015; right: *r*=0.256, *P*=0.001). Bilateral hippocampal volumes at 6 months of age were correlated with intracranial volume at 6 months of age (left: *r*=0.432, *P*=0.009; right: *r*=0.499, *P*=0.002), but not with birth weight (all *P*>0.150). No other variables listed in Table 1 significantly correlated with maternal anxiety and bilateral hippocampal volumes at both time points (*P*>0.1).

### Relations between maternal anxiety and hippocampus of infants

The regression models used for all analyses at each time point controlled for monthly household income, post-conceptual age on the MRI day and the corresponding intracranial volume. The regression models used for all analyses to examine hippocampal growth controlled for the time interval between the baseline and follow-up MRI scans, monthly household income and the growth of intracranial volume in the first six months of life. Note that in order to increase statistical power we did not control for birth weight, which was highly correlated with intracranial volume at birth (*r*=0.542, *P*<0.001).

**Figure 2:**
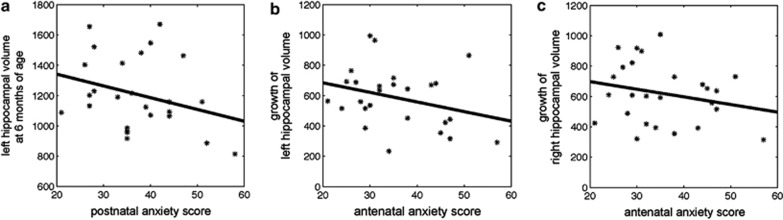
Scatter plots of the postnatal maternal anxiety score with the left hippocampal volume at 6 months of age (**a**), the antenatal maternal anxiety score with a growth in the left (**b**) and right (**c**) hippocampal volumes in the first 6 months of life.

The findings in Table 2 (top row) indicate that antenatal maternal anxiety was not associated with the bilateral hippocampal volumes of infants either at birth or at 6 months of age. This finding was not affected by controlling for postnatal maternal anxiety. However, the children of mothers that were more anxious during pregnancy show slower bilateral hippocampal growth in the first 6 months of life (Figures 2b and c). After controlling for postnatal maternal anxiety, the effect of antenatal maternal anxiety on the left hippocampal growth was no longer statistically significant. In contrast, after controlling for postnatal maternal anxiety the effect of antenatal maternal anxiety on the right hippocampal growth became stronger (*ß*=–0.477 vs −1.420), suggesting an independent contribution of antenatal maternal anxiety upon the right hippocampal development in early postnatal life.

The findings (Table 2, middle row) also indicate that the children of mothers with increased anxiety during the early postnatal period show significantly smaller left hippocampal volume at 6 months of age (Figure 2a). This effect of postnatal anxiety remained after controlling for antenatal maternal anxiety. Interestingly, the children of mothers with increased postnatal anxiety show greater right hippocampal growth in the first 6 months of life, however, this relationship was only significant after controlling for antenatal maternal anxiety.

**Figure 3:**
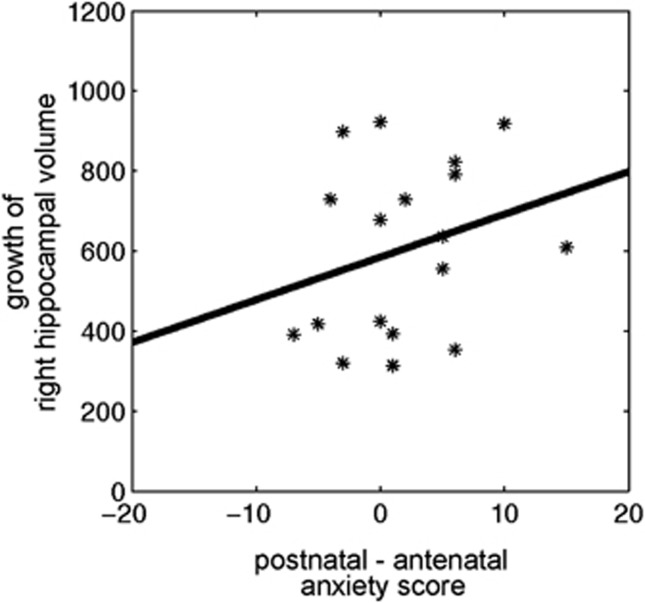
The association of the differential perinatal maternal anxiety scores with the growth of the right hippocampal volume in the first 6 months of life.

We then analyzed the hippocampal volume data as a function of the relation between antenatal and postnatal maternal anxiety (Table 2, bottom row; Figure 3). This analysis revealed that the children of mothers who reported an increase in anxiety levels from pregnancy to the postnatal period show significantly greater right hippocampal growth in the first 6 months of life.

## Discussion

This study examined the relation between antenatal and postnatal maternal anxiety and offspring hippocampal development over the first 6 months of life. We found that antenatal maternal anxiety did not influence bilateral hippocampal volume at birth. Instead, children of mothers reporting increased anxiety during pregnancy showed slower growth of both the left and the right hippocampus over the first 6 months of life. This effect of antenatal maternal anxiety becomes statistically stronger when controlling for levels of postnatal maternal anxiety. This finding reflects a strong positive association between postnatal maternal anxiety and the growth of the right hippocampus, with no discernible effect in the left hemisphere. Hence, postnatal growth patterns of the left and right hippocampi show distinct responses to postnatal maternal anxiety. The size of the left hippocampus during early development is likely to reflect the influence of both antenatal and post-maternal anxiety, whereas the growth of the right hippocampus is constrained by antenatal maternal anxiety, but enhanced in response to increased postnatal maternal anxiety.

Recently, elevated maternal cortisol concentrations during late pregnancy have been found associated with reduced fetal brain growth as measured by ultrasound,^24^ underscoring the importance of the intrauterine environment. As anxiety associates with elevations in cortisol levels,^25^ these findings are consistent with the effect of antenatal anxiety on hippocampal growth. Studies of antenatal maternal mood also reveal effects on child cognitive–emotional function even after controlling for postnatal maternal mood, suggesting a specific effect of antenatal maternal dysphoria.^26, 27^ Moreover, experimental studies with rodents and non-human primates suggest that maternal distress or glucocorticoid treatment during gestation can alter the development of limbic structures, including reduced hippocampal volume, with accompanying increases in fearfulness and increased stress reactivity.^28, 29^

In contrast, postnatal maternal anxiety associated with increased right hippocampal growth without any effect on the growth of the left hippocampus. However, postnatal maternal anxiety was negatively associated with the left hippocampal volume of 6-month-old infants, presumably reflecting a continuous influence of maternal anxiety across early development. Interestingly, smaller left hippocampal volume has been previously reported in relation to childhood trauma both in adults with major depression or post-traumatic stress disorder.^30, 31^ In pediatric research, findings about hippocampal volume in traumatized children are inconsistent. Most studies assessing children find no change in hippocampal volume after exposure to psychological trauma.^32, 33, 34^ However, Andersen *et al.*^35^ reported decreased hippocampal volume to be associated with sexual abuse at ages of 3–5 years and 11–13 years. Carrion *et al.*^36^ also found post-traumatic stress disorder symptoms and baseline cortisol related to hippocampal reduction over an ensuing 12- to 18-month interval in children with a history of maltreatment. The inconsistency concerning the presence of effects may partially be due to demographic differences across studies, such as age of trauma onset and level of psychopathology.^37^ Moreover, early maternal behavior significantly influences stress response and brain development in children.^38^ A majority of work examining parenting and infant development hypothesizes that poor parenting will result in neuroanatomical and neurocognitive deficits during childhood and later life.^38, 39, 40, 41^ However, in keeping with ideas concerning early-life programming and maturation,^42^ it is also reasonable to consider that experience in early development can influence neuronal growth, such that infants exposed to relatively poor parenting may invest a greater proportion of neural resources specifically in brain regions like the hippocampus, essential for stress regulation.^43^ Hence, variation in maternal behavior across these different studies could be another potential source for such discrepancies.

Genetic variation may also have an explanatory role, and genetic variation across studies may account for some of the aforementioned discrepancies in past research. For instance, brain-derived neurotrophic factor variation has been linked to both the presence of anxiety disorders in females^44^ and associated with alterations in hippocampal volume.^45^ Likewise, the short form of the 5HTTLPR serotonin promoter is associated with the presence of anxiety disorders (for example, generalized anxiety disorder, post-traumatic stress disorder and social anxiety disorder) as well as related traits,^46^ sensitivity to environmental adversity^47^ and attentional biases to threat.^48^ It was also directly related to smaller hippocampal volume in women, as well as in men who have additionally experienced childhood adversity.^49^ Taken together, such research may imply that the transmission of brain-derived neurotrophic factor, 5HTTLPR (or other genetic variants) from the mother to the child may help to explain our observed relation between maternal anxiety and infant hippocampal development. This possibility does not preclude the potential role of the postnatal environment, as the anxiety-relevant phenotypes may be most apparent when environmentally sensitive individuals repeatedly experience perceived threats and adversity.

The hemispheric-specific effect of postnatal maternal anxiety contrasts with the more extensive influence of antenatal maternal anxiety. The effect on the growth of the right hippocampus is consistent with the finding that this region is selectively associated with stress reactivity.^17^ Individual differences in the right hippocampal volume predict increased stress reactivity. Likewise, Rusch *et al.*^50^ show that trait anxiety is positively related to a larger right hippocampus in both depressed and normal subjects. Hence, the right hippocampal growth is enhanced in response to increased postnatal maternal anxiety, even though it is constrained by antenatal maternal anxiety. However, the size of the left hippocampus during early development is likely to reflect the influence of the exposure to perinatal maternal anxiety. It would serve as direct evidence in support of a major neuroanatomical theory of anxiety, that is, that the hippocampal formation is crucial in the development of anxiety.^51^

There are limitations in the study. Although the sample size at the baseline was large, it was relatively small at the follow-up study due to difficulties in imaging infants at 6 months of age. Hence, the results require replication. Antenatal maternal anxiety was only assessed at one time point during pregnancy. However, the second and third trimesters during pregnancy are critical periods when neural migration and synaptogenesis of the fetal brain rapidly develop. Considering both scientific importance and subjects' burden, our study only assessed antenatal maternal mood at the 26th week of gestation. Moreover, our assessment of maternal mood was based on a common screening tool intended to elicit a subjective report of emotional well being, but does not constitute a clinical assessment. The variations in hippocampus structure in the offspring are thus best considered as being associated with self-reported anxiety symptoms and not with clinical anxiety *per se*. Last but not least, our study did not incorporate potential influences of early maternal behavior and genetic variations on the brain development, which needs further investigation.

The major conclusion of this study is that the neuroimaging correlates of the familial transmission of phenotypes associated with anxiety-related disorders are apparent during early brain development. Furthermore, the level of maternal anxiety during pregnancy and in early postnatal life in our sample was in the normal range. We showed that relative antenatal and postnatal maternal influences might have a large societal impact. As such, we should optimize maternal perinatal health for all women and not only those at risk for maternal anxiety.

## Figures and Tables

**Table 1:** Demographics

	*Baseline*	*Follow-up*
	(n*=175)*	(n*=35)*
Gestational age (week), mean (s.d.)	38.7 (1.2)	38.5 (1.2)
Birth weight (gram), mean (s.d.)	3108.7 (405)	3121.0 (394)
Gender, male/female	92/83	20/15
Maternal anxiety, mean (s.d.)	38.2 (9.3)	37.2 (9.0)
Post-conceptual age on the MRI day (week), mean (s.d.)	40.1 (1.2)	66.4 (1.9)
Intracranial volume (cm^3^), mean (s.d.)	547.2 (47.2)	1013.9 (89.9)
Left hippocampal volume (mm^3^), mean (s.d.)	593.5 (67.7)	1190.4 (223.7)
Right hippocampal volume (mm^3^), mean (s.d.)	636.3 (74.6)	1264.2 (219.7)
*Ethnicity, %*		
Chinese	42.4	31.4
Malay	41.9	57.2
Indian	15.7	11.4
*Monthly*		
⩽999	3.4	5.7
*Household Income*		
1000–1999	14.3	17.1
(S$), %*		
2000–3999	40.0	28.6
4000–5999	21.1	22.9
⩾6000	13.1	17.1
Unreported	8.0	8.6
Prenatal smoking exposure, % yes	54.45	47.1
Prenatal alcohol exposure, % yes	1.7	0

*P<0.05.

**Table 2:** Effects of perinatal maternal anxiety on the hippocampal volumes of infants at birth and 6 months as well as the hippocampal growth volume in the first 6 months of infants' life

	*At birth*^a^		*At 6 months*^b^		*Growth in the first 6 months of life*^c^	
	*LH*	*RH*	*LH*	*RH*	*LH*	*RH*
*Antenatal anxiety*						
No control for postnatal anxiety	−0.067	−0.036	−0.355	−0.225	−0.544*	−0.477*
Control for postnatal anxiety	—	—	0.172	−0.197	−0.358	−1.420**
*Postnatal anxiety*						
No control for antenatal anxiety	—	—	−0.382*	−0.134	−0.346	−0.089
Control for antenatal anxiety	—	—	−0.629*	−0.039	−0.201	0.992*
Postnatal -antenatal anxiety	—	—	−0.318	0.005	−0.049	0.620*

Standardized β values are listed in the table.

Abbreviations: LH–left hippocampus; RH–right hippocampus.

*P<0.05; **P<0.01.

a Model adjusted for age at the baseline MRI, household income and intracranial volume at birth.

b Model adjusted for age at the follow-up MRI, household income and intracranial volume at 6 months.

c Model adjusted for age difference between the baseline and follow-up MRI, household income and growth of intracranial volume in the first 6 months of life.
